# A community-engaged randomized controlled trial of an integrative intervention with HIV-positive, methamphetamine-using men who have sex with men

**DOI:** 10.1186/s12889-016-3325-1

**Published:** 2016-07-30

**Authors:** Adam W. Carrico, Jennifer Jain, Michael V. Discepola, David Olem, Rick Andrews, William J. Woods, Torsten B. Neilands, Steven Shoptaw, Walter Gómez, Samantha E. Dilworth, Judith T. Moskowitz

**Affiliations:** 1 Department of Public Health Sciences, University of Miami School of Medicine, 1120 NW 14th St., Office 1005, Miami, FL 33136 USA; 2 University of California, San Francisco School of Nursing, San Francisco, USA; 3 San Francisco AIDS Foundation, San Francisco, USA; 4 University of California, San Francisco Center for AIDS Prevention Studies, San Francisco, USA; 5 Departments of Family Medicine and Psychiatry, University of California, Los Angeles David Geffen School of Medicine, Los Angeles, USA; 6 Department of Medical Social Sciences, Northwestern University, San Francisco, USA

**Keywords:** Contingency management, Resilience, Men who Have Sex with men, Methamphetamine, HIV/AIDS, Positive affect

## Abstract

**Background:**

Contingency management (CM) is an evidence-based intervention providing tangible rewards as positive reinforcement for abstinence from stimulants such as methamphetamine. Integrative approaches targeting affect regulation could boost the effectiveness of CM in community-based settings and optimize HIV/AIDS prevention efforts.

**Methods/Design:**

This randomized controlled trial with HIV-positive, methamphetamine-using men who have sex with men (MSM) is examining the efficacy of a 5-session, individually delivered positive affect regulation intervention – Affect Regulation Treatment to Enhance Methamphetamine Intervention Success (ARTEMIS). ARTEMIS is designed to sensitize individuals to non-drug-related sources of reward as well as assist with managing depression and other symptoms of stimulant withdrawal during CM. HIV-positive, methamphetamine-using MSM who are enrolled in a community-based, 12-week CM program are randomized to receive ARTEMIS or an attention-matched control condition. Follow-up assessments are conducted at 3, 6, 12, and 15 months after enrollment in CM. Four peripheral venous blood samples are collected over the 15-month follow-up with specimen banking for planned biomarker sub-studies. The primary outcome is mean HIV viral load. Secondary outcomes include: sustained HIV viral suppression, T-helper cell count, psychological adjustment, stimulant use, and potentially amplified transmission risk behavior.

**Discussion:**

Implementation of this randomized controlled trial highlights the importance of delineating boundaries between research activities and community-based service provision. It also provides insights into best practices for integrating the distinct agendas of academic and community partners in clinical research. This trial is currently enrolling and data collection is anticipated to be completed in September of 2018.

**Trial registration:**

This trial was registered on clinicaltrials.gov (NCT01926184) on August 16, 2013.

## Background

There is increasing recognition that individuals who use stimulants such as cocaine, crack, or methamphetamine experience profound HIV-related health disparities [1]. HIV-positive men and women who use stimulants display substantially elevated HIV viral load, which appears to be primarily due to difficulties with anti-retroviral therapy (ART) adherence and persistence [2–4]. Consequently, HIV-positive men and women who engage in more frequent stimulant use are at elevated risk for faster HIV disease progression [5–7]. The co-occurrence of elevated HIV viral load and HIV transmission risk behavior among HIV-positive, stimulant-using men who have sex with men (MSM) also results in amplified risk of onward HIV transmission [8, 9]. As a result, there is increasing interest in testing substance abuse interventions with HIV-positive, stimulant-using MSM with the goal of increasing rates of sustained HIV viral suppression to optimize the effectiveness of HIV treatment as prevention [10, 11].

Contingency management (CM) is an evidence-based, behavioral intervention where individuals receive tangible reinforcement for biologically confirmed substance abstinence that has been tested as a stand-alone intervention and shown to boost the effectiveness of cognitive-behavioral therapy with stimulant users [12, 13]. Although randomized controlled trials (RCTs) provide support for the efficacy of CM for decreasing stimulant use among substance-using MSM [14, 15], the effects are moderate and not consistently sustained over long-term follow-up. Similarly, a previous RCT with HIV-positive opiate and cocaine users observed decreases in HIV viral load as well as HIV risk behaviors immediately following CM, but these were not maintained over follow-up [16]. These short-term benefits may be partially attributable to the difficulties individuals can experience with achieving abstinence during CM [17] and the underscore the need for more intensive treatment with those who have chronic, relapsing substance use disorders [18]. In San Francisco, the Department of Public Health has been successfully implementing a 12-week CM intervention with methamphetamine-using MSM since 2004 as one component of more comprehensive substance abuse treatment services tailored for MSM [19–21].

Novel approaches are needed to assist methamphetamine-using MSM with managing symptoms of stimulant withdrawal that potentially undermine the effectiveness of CM. Negative reinforcement models of addiction [22] highlight that increases in depression and other forms of negative affect during withdrawal are a potent, chronic stressor that can serve as a trigger for stimulant use in MSM [23]. Consequently, interventions that provide coping skills training and promote psychological resilience could boost and extend the effectiveness of CM. Informed by Revised Stress and Coping Theory, positive affect (e.g., happiness, gratitude) may reinvigorate coping efforts in the midst of chronic stress and build supportive social networks that assist individuals with managing stressful circumstances [24, 25]. Lending support to its potential adaptive significance, positive affect is associated with decreased sexual risk taking behavior in MSM and enhanced HIV disease management [26–28]. These findings are generally consistent with the beneficial associations of positive affect with health and well-being in other populations [29], but important questions remain regarding whether this reflects personality factors or if the experience of more frequent positive affect is linked to better outcomes [30].

Positive affect interventions could boost the effectiveness of existing approaches to promote health behavior change, and more clinical research is needed with HIV-positive substance users. One RCT with hypertensive African Americans observed that a positive affect intervention delivered with patient education increased medication adherence at 12 months compared to patient education alone, but no concurrent intervention-related effects on blood pressure were observed [31]. Similarly, another RCT with patients receiving a percutaneous coronary procedure observed that a positive affect intervention delivered with patient education increased physical activity by almost 2-fold at 12 months compared to patient education alone [32]. Among those with alcohol and substance use disorders, positive affect interventions may increase responsivity to non-substance-related sources of reward, sustain cognitive-behavioral change processes that are relevant to recovery, and help build social relationships that are crucial to reducing or avoiding substance use [33, 34]. One pilot RCT of a web-based gratitude intervention with individuals receiving outpatient treatment for an alcohol use disorder observed short-term increases in low activation positive affect compared to an attention-control condition [35]. We also completed a pilot RCT with methamphetamine-using MSM where a positive affect intervention delivered during CM achieved short-term increases in positive affect compared to CM alone [36]. Building upon this formative clinical research, we present the protocol for a Phase II RCT of a positive affect intervention designed to serve as an adjuvant to CM with HIV-positive, methamphetamine-using MSM.

## Method and design

This Phase II RCT is examining the efficacy of a 5-session positive affect intervention that is designed to boost and extend the effectiveness of CM with HIV-positive, methamphetamine-using MSM (www.clinicaltrials.gov; NCT01926184). All enrolled participants are referred to a community-based, 12-week CM program that must be initiated prior to randomization. CM visits are completed at the San Francisco AIDS Foundation and all other trial-related activities occur at a separate field site in the community. Screening and enrollment are anticipated to continue through June of 2017 and follow-up assessments will be completed in September of 2018. All relevant procedures are approved by the Institutional Review Boards for the University of California, San Francisco and Northwestern University, and this RCT received a certificate of confidentiality from the National Institute on Drug Abuse. The data safety and monitoring plan is overseen by the University of California, Los Angeles Data Safety and Monitoring Board for Addiction Medicine, which holds annual meetings to review participant-related events and overall progress for this RCT.

### Recruitment, screening, and enrollment for the RCT

HIV-positive, methamphetamine-using MSM are recruited for this RCT through three primary sources. First, men initiating services at a community-based CM program complete a brief consent form to be contacted by study staff to learn more about the RCT. Second, direct recruitment is conducted using flyers and palm cards that are distributed in HIV medical clinics, AIDS service organizations, bars and clubs, bath houses, and via social media. Third, an incentivized snowball sampling method is employed where eligible participants receive a maximum of $30 for referring up to three individuals who are subsequently judged to be eligible for the RCT.

To be eligible for this RCT, participants must meet the following inclusion criteria: 1) 18 years of age or older; 2) identify as a MSM; 3) provide documentation of HIV-positive serostatus (i.e., letter of diagnosis or ART medications other than Truvada that are matched to their photo identification); and 4) provide a urine or hair sample that is reactive for methamphetamine metabolites. All potentially eligible participants referred from the community-based CM program are scheduled for an in-person screening visit. Participants recruited from other sources are required to report methamphetamine use in the past 30 days to be eligible for an in-person screening visit.

At the in-person screening visit, participants complete a signed informed consent, consent for specimen banking, a detailed tracking form with at least one secondary contact, and a consent to search relevant death registries. Participants also complete a Health Insurance Portability and Accountability Act (HIPAA) release to access treatment records at the CM program. Those who provide a urine sample that is reactive for methamphetamine or have documented evidence of a reactive urine screen for methamphetamine from the CM program are immediately scheduled for a baseline assessment. Those without evidence or recent methamphetamine use from urine screening provide a hair sample for toxicology testing. Hair is collected from the occipital region of the scalp where possible, but it is also collected from other sites (i.e., beard, body) where necessary to obtain biological confirmation of recent methamphetamine use. After the screening visit, participants receive a $50 pre-loaded debit card. Participants are excluded after the screening visit for the following reasons: 1) prescription of a psychostimulant (e.g., Adderall) that cross reacts with urine screening for methamphetamine; 2) inability to provide informed consent; 3) unable to follow the study protocol; and 4) judged by the principal investigators that it is not in the best interests of the individual to participate.

### Community-based CM program

This RCT is being conducted in partnership with a community-based, 12-week CM program for methamphetamine-using MSM that is operated by the San Francisco AIDS Foundation. Individuals who are interested in the CM program complete a detailed intake that includes assessments of the frequency with which they use methamphetamine and engage in sexual behaviors with men. MSM who report using methamphetamine at least weekly on average in the past three months provide a urine sample for toxicology screening at the intake visit and are enrolled in the CM program irrespective of urine toxicology results.

All clients in the CM program are encouraged to pursue abstinence from methamphetamine and other stimulants during the 12-week CM period. Urine sample collection is directly observed by CM program staff. The voucher for the initial sample that is negative for methamphetamine and cocaine metabolites is worth $2.00. Vouchers increase in value by 25 cents for each consecutive stimulant-free sample to a maximum of $10.00. Participants earn an $8.50 bonus voucher for every third consecutive stimulant-free sample. A rapid reset procedure allows participants to return to their place in the escalating reinforcement schedule after producing three consecutive urine samples that are non-reactive for methamphetamine and cocaine [19]. The total possible reinforcement is $330, and participants may choose to receive incentives earned any time during or after the 12-week CM intervention period.

Concurrent with the CM urine screening visits, clients may participate in a drop-in group that provides social support for continuing to pursue goals for stimulant use reduction or abstinence. Drop-in groups also provide opportunities to facilitate linkage to community-based services including formal substance abuse treatment and HIV care. After completing the 12 weeks of urine screening visits, individuals are invited to attend aftercare sessions to continue to forge social networks that are supportive of avoiding stimulant use and develop relapse prevention plans. Those who complete the 12-week CM program with aftercare are invited to continue attending regular drop-in groups to provide support and mentorship to men who are actively completing the thrice weekly urine screening visits.

### Run-in period and randomization

All eligible participants complete a run-in period where they are required to complete five separate visits prior to randomization. These visits include: 1) a baseline assessment with a peripheral venous blood sample; 2) three urine screening visits (regardless of the toxicology results) at the community-based CM program; and 3) a separately scheduled randomization visit where the first intervention or attention-control session is administered. Participants who fail to complete the run-in period are not randomized and do not complete subsequent visits for this RCT unless at some point they re-initiate contact with the study team and choose to be re-screened. Randomization is accomplished using a computer generated sequence with randomly permuted block sizes of 2, 4, and 6 to guard against subversion. Only the study data manager has access to the computer-based randomization algorithm. Because randomization occurs at the first intervention or attention-control session, this ensures that all participants in this RCT complete at least one of the five individually delivered sessions.

### Positive affect intervention

Affect regulation treatment to enhance methamphetamine intervention success (ARTEMIS) is a multi-component, individually-delivered 5-session intervention targeting positive affect regulation. This intervention was adapted from prior clinical research testing the feasibility and acceptability of a positive affect intervention for recently diagnosed HIV-positive persons [37]. The extant positive affect intervention protocol was adapted and pilot tested in a RCT with 21 methamphetamine-using MSM receiving CM [36]. Based on the results of the pilot RCT, the positive affect intervention protocol was further refined prior to initiating the present Phase II RCT with HIV-positive, methamphetamine-using MSM.

The original positive affect intervention protocol consists of eight skills:

(1) positive event noting; (2) positive event capitalizing; (3) gratitude; (4) informal and formal mindfulness; (5) positive reappraisal; (6) personal strengths; (7) attainable goals; and (8) acts of kindness. As shown in Table 1, we modified the original protocol to tailor this positive affect intervention for HIV-positive, methamphetamine-using MSM. These adaptations included additional content to optimize delivery of the core positive affect regulation skills with this population and new components designed to facilitate greater engagement in the recovery process (e.g., problem-focused coping, values clarification, and referral to community-based services). Informed by prior research examining the efficacy of mindfulness-based relapse prevention [38–40], participants also complete meditation exercises during ARTEMIS intervention sessions to further enhance metacognitive awareness and assist individuals in coping more effectively with methamphetamine withdrawal. Self-report measures of stimulant craving before and after each session as well as a measure of positive and negative affect before sessions one, three, and five are completed by participants. Participants can choose to receive $20 in cash per session or two $50 debit cards upon completion of all five sessions, complete detailed home practice exercises, receive a workbook with each of the skills outlined and an iPod shuffle that is pre-loaded with meditation exercises to facilitate regular practice.

**Table 1:** Positive affect intervention protocol for HIV-positive, methamphetamine-using MSM

Session	Positive Affect Regulation Skills	Additional Intervention Content
1	Noticing Positive Events	Psychoeducation on Stimulant Withdrawal
	Capitalizing on Positive Events	Capitalizing on Non-Reactive Urine Toxicology
	Gratitude	Breathing Retraining with Positive Event Imagery
2	Mindfulness (Informal and Formal)	Breath Meditation
		Self-Compassion
3	Positive Reappraisal	Problem-Focused Coping & Reasoned Action
		Breath Meditation
4	Strengths	Values Clarification
	Attainable Goals	Mountain Meditation
5	Altruism	Volunteer opportunities
		Linkage to More Intensive Community-Based Services
		Loving Kindness Meditation

Session one begins with an overview of the intervention and psychoeducation about the stressful nature of stimulant withdrawal that individuals experience during CM. Positive affect regulation skills are framed as one way of more effectively coping during this stressful period. Participants are encouraged to start by noticing positive events that occur in daily life and explore ways to amplify or extend the experience of positive emotions related to these events (i.e., capitalizing). In addition to identifying positive events in their daily lives, participants are asked to describe positive emotions that stem from having a urine screen during CM that was non-reactive for stimulants (where applicable) and continue to capitalize on this positive event. The concept of gratitude as a feeling of appreciation or thankfulness that can stem from positive events is also introduced. Finally, facilitators deliver a modified breathing retraining exercise that includes positive event imagery to assist participants with capitalizing on a positive event in vivo. For home practice, participants are asked to complete the “One Good Thing” exercise where they note a positive event that happened each day and briefly describe responses to the event. Participants are also asked to begin keeping a gratitude journal that continues throughout the 5-session intervention [41].

Session two includes mindfulness-based skills training to cultivate non-judgmental, present-centered awareness. By facilitating metacognitive awareness, mindfulness promotes greater insight into cognitive and emotional responses to events which may reduce craving and decrease the likelihood that individuals engage in the overlearned response of substance use [39]. Informal mindfulness exercises are completed in-session to encourage individuals to develop an enhanced awareness of daily experiences (e.g., brushing one’s teeth). Participants also complete a brief formal mindfulness-based meditation practice (i.e., breath awareness) and receive an iPod shuffle preloaded with three meditation exercises: breath awareness, mountain meditation and loving kindness meditation [42]. Finally, this session concludes with a discussion of the importance of self-compassion as a way of coping with stressful life circumstances. Participants are encouraged to be more tolerant of their imperfections by developing a supportive and caring self-orientation. Home practice includes informal mindfulness practice (i.e., “Practicing Being Present in Daily Life” exercise), formal mindfulness meditation using the iPod shuffle, and the gratitude journal. Participants may listen to any of the three recordings on the iPod shuffle.

Session three focuses on executing effective coping responses. Participants are introduced to the concept of positive reappraisal whereby individuals change their interpretations of stressful events to support successful adaptation. The facilitator reviews ways in which changing thoughts about a stressful event (i.e., positive reappraisals) can affect emotional responses and subsequent behaviors. This is generally consistent with the approach of cognitive-behavioral treatments like the Matrix Model [43], but there is no requirement that the initial appraisal of an event is distorted. Especially where aspects of a stressful event are uncontrollable, participants are encouraged to examine ways in which the experience could have been worse or how they learned something new as a result of enduring this stressful event (both examples of positive reappraisals). Participants are asked to identify a recent stressful event and work with the facilitator to practice different methods for positive reappraisal in session.

Another important component of session three is reviewing methods of enhancing coping effectiveness in response to controllable situations [44, 45]. Where aspects of stressful circumstances are controllable, participants are encouraged to explore problem-focused coping strategies that can be implemented to reduce the experience of stress. Participants briefly review possible problem-focused coping strategies with the facilitator. This process of executing effective coping responses by engaging in positive reappraisal and then problem-focused coping is presented as “reasoned action” where managing emotional responses to an event is a critical first step towards addressing any aspects that are within one’s control. Participants also complete a formal mindfulness-based meditation practice (i.e., breath awareness) at this session. Home practice includes daily positive reappraisal exercises where participants identify stressful events, develop positive reappraisal(s), and delineate concrete actions that can be taken to address the identified ‘problem.’ Participants also continue with the meditation practice and the gratitude journal.

Session four begins with an exercise where participants are asked to reflect on personal strengths. This process promotes greater insight into personal sources of resilience and enhances self-affirmation, which has been linked to enhanced problem solving and self regulation [46, 47]. After identifying personal strengths, participants are asked to examine the degree to which these strengths are linked to personal values or the ways in which strengths assist one with living values in action. Then, participants are introduced to the concept of attainable goals, which are presented as short-term goals that are Achievable, have Clear steps, and a definitive Endpoint (ACE). Strengths and values are presented as important resources that energize goal-oriented progress. Participants are encouraged to examine ways in which leveraging personal strengths could facilitate progress toward attainable goals. Values are framed as the underlying meaning structures that remind individuals why attainable goals are important. This session concludes with the mountain meditation which focuses on embracing personal strengths and resilience in the midst of stress [42]. For home practice, participants are asked to monitor progress towards completing attainable goals identified in session while noting personal strengths that assist with progress toward these goals. Participants also continue with the meditation practice and the gratitude journal.

Session five targets altruism by discussing findings that small acts of kindness may enhance psychological adjustment and bolster physical health [48, 49]. Participants review ways in which they can incorporate small acts of kindness into their daily activities and review formal volunteer opportunities available in the community. Participants also complete the loving kindness meditation [42], which focuses on experiencing feelings of love and caring toward another person. The facilitator also leads the participant through a thorough skills review of all the positive affect and coping skills delivered throughout the five sessions. This portion of the intervention focuses on discussing positive aspects of the ARTEMIS sessions for the participant and ways in which he can continue to pursue treatment in the community. Tailored referrals are provided to assist participants with engaging in more intensive mental health or substance abuse treatment.

### Attention-control condition

The attention-control consists of five sessions that include face-to-face administration of psychological measures and neutral writing exercises [50]. Participants are instructed to write as if they were reporting facts without going into any of the thoughts or feelings about the events (e.g., plans for the next 24 h). All sessions are comparable in length to the intervention sessions, but do not include any skills practice. Self-report measures of stimulant craving before and after each session as well as a measure of positive and negative affect before sessions one, three, and five are completed by participants. Participants can choose to receive $20 cash for completing each attention-control session or two $50 debit cards upon of the completion of the fifth session, and an iPod with three preloaded pop songs.

### Staff training and fidelity monitoring

Each staff member receives a detailed training on assessment protocols for this RCT: 1) privacy and confidentiality including HIPAA security practices and human subjects protection training; 2) obtaining informed consent; 3) blood borne pathogens training 4) safe shipping of biological specimens training 5) laboratory safety for researchers training 6) technology training including use of laptops for data collection and the participant database; 7) interviewing techniques such as building rapport, working with diverse populations, cultural sensitivity, population-specific issues and referrals, maintaining confidentiality, basic quantitative and qualitative interview skills; 8) a detailed suicidality assessment protocol; and 9) and role playing of assessments or individual sessions.

Intervention facilitators are provided with a detailed manual that describes the procedures for administering the five individual intervention sessions. Facilitators conducted mock sessions and the audio recordings of these sessions are reviewed by a clinical supervisor who then meets one-on-one with the facilitator to provide feedback on delivery of intervention content and process-oriented techniques. Mock sessions are repeated until the facilitator is judged to be competent with each of the five intervention sessions.

The protocol for monitoring the fidelity of the positive affect intervention sessions during the course of the study includes audio recording of sessions for weekly review with a clinical supervisor and fidelity checklist ratings that are reviewed monthly in group supervision. Audio recordings of intervention sessions are reviewed by the fidelity monitor and the clinical supervisor to assess adherence to the protocol, delivery, interpersonal skills, rapport, and session flow. Approximately 10 % of all positive affect intervention sessions are coded using fidelity rating checklists with detailed feedback provided to facilitators.

### Follow-up assessments and specimen banking

At 3, 6, 12 and 15 months after enrollment in CM, each participant completes computer-based follow-up assessments that are administered by a trained interviewer who has not administered intervention or attention-control sessions to that participant. Self-reported substance use and sexual behavior measures are completed by participants using audio computer-assisted self-interviewing to enhance reliability and validity [51]. Urine samples for on-site toxicology testing for recent use of methamphetamine, cocaine, opiates, marijuana, and benzodiazepines (Redwood Biotech iCup®; Santa Rosa, CA) are collected at each assessment. Peripheral venous blood samples are collected at baseline as well as 6, 12, and 15 months following enrollment in CM to measure HIV viral load and CD4+ T-cell count. Participants receive a pre-loaded $50 debit card for completing each of the assessment visits over the 15-month period.

Participants are also asked to provide an additional 25 ml of peripheral venous blood using two 10 mL EDTA and two 5 mL PAXgene® tubes (Qiagen, Inc.) at baseline as well as 6, 12, and 15 months following enrollment in CM. Using EDTA tubes, leukocyte DNA is extracted and banked. Approximately three mL of plasma are also banked. PaXgene® tubes are used for isolation and stabilization of RNA. These tubes are progressively frozen and banked.

### Primary and secondary outcome measures

#### HIV viral load

HIV viral load testing is performed to detect plasma HIV RNA using the Abbott RealTime HIV-1 assay (Abbott Molecular, Inc.; Des Plaines, IL). Although this assay has a lower limit of detection of 40 copies/mL, valid viral load values below this level are observed with less reliable frequency [52]. The primary outcome for this RCT is change in mean log_10_ HIV viral load. Sustained HIV viral suppression, defined as an undetectable HIV viral load (<40 copies/mL) over the 15-month follow-up, will be examined as a secondary outcome.

#### CD4+ T-cell count

CD4+ T-cell count is measured with whole blood using flow cytometry. CD4+ assays are performed by Quest Diagnostics.

#### ART adherence and persistence

At each assessment, participants currently taking ART medications are asked to rate their adherence to each medication during the past 30 days using the visual analogue scale [53]. Participants are also asked to indicate if they have experienced a treatment interruption, a period of two days or more where all HIV medications were stopped without guidance from an HIV primary care provider. Using these measures, a composite index of ART adherence and persistence will be calculated by summing the following indicators (1 point each): 1) currently on ART; 2) no treatment interruption in the past six months; and 3) 100 % self-reported ART adherence in the past 30 days.

#### Psychological adjustment

The Differential Emotions Scale (DES) is administered to assess the frequency of positive and negative affect during the past week [54]. Participants rate how frequently they felt a particular emotion during the past week. The DES measures high and low level intensity positive and negative affect, which will be examined as separate outcomes. The Center for Epidemiological Studies Depression Scale (CES-D) is administered to measure symptoms of depression during the past week [55, 56].

#### Stimulant craving

The Penn Alcohol Craving Scale is a five-item self-report measure that was adapted for assessment of methamphetamine craving [57]. Frequency, intensity, and duration of thoughts about using methamphetamine are assessed at each assessment visit. Immediately before and after each individual session (i.e., ARTEMIS intervention or attention-control), participants also rate the intensity of their current craving for methamphetamine, powder cocaine, and crack cocaine separately using a visual analogue scale from zero (no craving at all) to 100 (some of the worst craving ever).

#### Stimulant use

Participants report how often they have used various substances in the past three months. Participants who have used a given substance in the past three months are asked to report the number of days they used that substance in the past 30 days, which is consistent with Addiction Severity Index [58]. Participants also report the longest number of days in a row they used each substance in the past 30 days to index binge use [59]. Finally, those who provide a urine sample that is reactive for methamphetamine or cocaine (1) will be compared to those who provide a urine sample that is not reactive for these stimulants (0).

#### Potentially amplified transmission risk behavior

Participants report the number of anal sex partners in the past 3 months, stratified by whether or not they were feeling the effects of methamphetamine [21]. The total number of condomless HIV-negative or unknown serostatus partners is calculated, stratified by methamphetamine use. Potentially amplified transmission risk partners are the number of condomless anal sex partners who are HIV-negative or unknown serostatus where participants have a HIV viral load greater than 200 copies/mL at the same assessment visit. The total number of potentially amplified transmission risk partners stratified by methamphetamine use will be calculated. We will also examine any engagement in any potentially amplified transmission risk as an outcome.

### Outcome analyses

We will first generate one-way frequency tables for all variables and measures of central tendency and variability for continuous variables (e.g., means, medians, standard deviations) to perform range checks, quantify the amount of missing data, and yield valuable descriptive findings. Chi-square tests, *t*-tests, and comparable two-group non-parametric tests will be used to investigate equality of potential confounders (e.g., homelessness) across the intervention and attention-control conditions at baseline. Similarly, we will confirm that there are no differences in the mean number of intervention and attention-control sessions completed as well as no differential attrition by study arm.

Based on our extensive experience working with this population, we anticipate a maximum of 20% attrition over the 15-month investigation period. Attrition analyses will compare respondents who complete all measurements to those who do not based on baseline characteristics and we will test for differential attrition by intervention condition. Direct maximum likelihood and multiple imputation will be used to address incomplete data because these methods make the relatively mild assumption that missing data arise from a conditionally missing-at-random process [60].

Intent-to-treat analyses will be conducted primarily using multilevel random coefficient models (i.e., hierarchical linear modeling; HLM). These models incorporate random intercepts and slopes for each participant based on the participant’s multiple measurements over time [61]. Initial models will be unadjusted and compare outcome trajectories for participants in ARTEMIS + CM and Attention-Control + CM across time via the group-by-time interaction effect. If preliminary analyses detect imbalance in potential confounders across the groups that cannot be satisfactorily addressed via covariate adjustment, we will substitute causal inference methods based on the Rubin causal model (e.g., propensity score weighting; marginal structural models) to obtain population-level effects of the intervention under the assumption of balanced confounders between the two groups [62–66]. A priori planned comparisons will be performed to test group differences at 3, 6, 12, and 15 months for both the primary and secondary outcomes. These planned comparisons will be evaluated at α = .05; any subsequent post-hoc comparisons will be adjusted via simulation-based stepdown methods to maintain a nominal Type I error rate of .05 [67].

The primary outcome will be log_10_ HIV viral load over the 15-month follow-up. Sustained HIV viral suppression will be examined as a secondary outcome using logistic regression of the binary outcome of sustained virologic suppression over the 15-month follow-up (0 = no; 1 = yes) on randomization group assignment. Other continuous secondary outcomes that will be examined in intent-to-treat analyses include: CD4+ T-cell count, positive and negative affect, stimulant craving, and self-reported stimulant use. Ordered categorical and categorical outcomes assessed repeatedly over the 15-month follow-up include: ART adherence and persistence, a positive urine toxicology result for stimulants, potentially amplified transmission risk partners stratified by methamphetamine use, and any potentially amplified transmission risk.

### Power analysis

Using NCSS PASS with a total sample size of 150, 80 % retention, and four repeated measures of viral load, the minimum detectable effect sizes are in the small-medium range (Cohen’s *d* = 0.29–0.47). Estimates of the minimum detectable effect size vary depending on the within-subject correlation. Overall, this RCT has adequate power to detect moderate effects of ARTEMIS + CM on the primary outcome, log_10_ HIV viral load.

## Discussion

Informed by Revised Stress and Coping Theory, positive affect is thought to serve unique, adaptive functions in the midst of chronic stress because it sustains coping efforts and builds personal resources for coping with stress [24, 25]. Among those with alcohol or substance use disorders, positive affect may sensitize individuals to non-substance-related sources of reward, sustain cognitive-behavioral change processes that are relevant to recovery, and help build social relationships that are supportive reducing or avoiding substance use [33, 34]. There is also increasing recognition that positive affect is associated with HIV-related health behavior change [26–28], and positive affect interventions may optimize the effectiveness of health education in other patient populations [31, 32]. This RCT is among the first to test the efficacy of a positive affect intervention designed to boost and extend the effectiveness of CM with HIV-positive, methamphetamine-using MSM.

Prior research observed that CM is effective for reducing HIV viral load in the short-term [16], and integrative approaches may be needed to achieve sustained decreases in HIV viral load. By examining log_10_ HIV viral load as the primary outcome as well as sustained HIV viral suppression and CD4+ T-cell count as secondary outcomes, our team will be able to index whether a positive affect intervention delivered with CM can achieve meaningful improvements in HIV disease markers compared to CM alone. To our knowledge, only two prior RCT’s of cognitive-behavioral interventions with methamphetamine-using MSM observed reductions in condomless anal intercourse, which were modest and not consistently maintained over follow-up [15, 68]. Novel approaches for HIV-positive, methamphetamine-using MSM are needed to reduce viral load in this population in order to mitigate risk of onward HIV transmission and optimize health outcomes. In the present RCT, any effects of ARTEMIS + CM on HIV viral load could be attributable to the effects of enhanced psychological adjustment and reduced stimulant use on greater ART adherence and persistence. These mediating pathways will be examined to better understand any underlying mechanisms whereby ARTEMIS + CM may optimize the effectiveness of HIV treatment as prevention.

One important strength of this RCT is that it requires biological confirmation of recent methamphetamine use, which has not been part of the inclusion criteria for prior RCTs of CM with this population [14, 15, 17]. Biological confirmation of recent methamphetamine use maximizes internal validity by ensuring that participants are not merely reporting methamphetamine use to receive incentives from the CM program as well as study-related visits. On the other hand, it introduces concerns that community members may become aware of the inclusion criteria and use methamphetamine to be eligible. In order to successfully implement this RCT, our team has gone to great lengths to conceal inclusion criteria by not referring directly to methamphetamine use in any recruitment materials, conducting an extensive screening visit with multiple assessment measures to conceal inclusion criteria, and refraining from engaging discussions of the inclusion criteria with those who are not eligible. At the same time, requiring biological confirmation of recent methamphetamine use has presented challenges for achieving enrollment targets. We have been unable to collect hair from the scalp for some men to establish that they meet the inclusion criteria, and hair from other body sites is not an optimal indicator of recent methamphetamine use [69]. There are also individuals who have achieved early partial abstinence that are at risk of relapse, but we are unable to enroll these participants because we cannot obtain biological confirmation of recent methamphetamine use. Further advances in methamphetamine toxicology screening are needed to optimize clinical research with substance-using populations.

CM for this RCT is delivered by a community-based program, which has introduced a number of opportunities and challenges. The overall objectives of this RCT are substantially supported by leveraging an established and well respected CM program in the community for methamphetamine-using MSM. Specifically, our relationship with a community-based CM program substantially supported our efforts to refine and deliver the ARTEMIS intervention as well as enhanced our recruitment and retention efforts. At the same time, there are challenges with navigating the enrollment goals of this RCT with the relatively large number of men seeking CM services, only a subset of who are eligible for this RCT. Because the CM program strives to provide services on demand and without regard to HIV status, conducting this RCT has led to substantial increases in the number of men receiving services to maximize the number of participants who meet criteria for randomization. Due to the popularity of this CM program, we have experienced difficulties with reserving space for individuals who meet our inclusion criteria, thus creating a tension between the enrollment target for the RCT and clinical service delivery. Navigating these challenges has required ongoing discussions to integrate the distinct agendas of the academic and community partners.

Conducting a community-engaged RCT with a CM program has also required a greater focus on clearly delineating roles and responsibilities. Although participants complete a HIPAA release to allow for communications with the CM program, this is for the expressed purpose of establishing eligibility for randomization, to extract the CM urine screening results and CM incentives received from the clinical records, and assist with tracking for follow-up. Due to our close and ongoing interactions with the CM program, the research team must avoid discussions that are relevant to the clinical care participants are receiving in the CM program and strive to disclose only the minimum information necessary to CM program staff regarding each participant’s study activities. Similarly, our close connection with the CM program can create role confusion for participants that contributes to a therapeutic misconception [70]. This requires staff to have detailed and ongoing discussions with participants about the distinctions between participating in this RCT and receiving clinical services. Staff note their ethical obligation to ensure the safety of participants, conducting detailed screenings for suicidal or homicidal ideation, plan, and intent where necessary. However, participants often present with other acute stressors such as food insecurity and housing instability that are beyond the scope of the role of research staff. In these instances, participants are provided with a detailed referral sheet and encouraged to seek out clinical staff at the CM program where appropriate.

ARTEMIS acknowledges that not all HIV-positive, methamphetamine-using MSM are ready, willing, or able to abstain from stimulant use. The focus of ARTEMIS on positive affect and non-substance-related sources of reward challenges the dominant paradigms of addiction treatment because it does not directly target psychological processes such as motivation that are traditionally conceptualized as being most relevant to reducing substance use. Instead, the positive affect intervention is designed to build the capacity of participants to cope more effectively with depression and other symptoms of stimulant withdrawal, which could maximize success with stimulant abstinence during CM and boost the long-term effectiveness of CM.
